# Long noncoding RNAs in DNA methylation: new players stepping into the old game

**DOI:** 10.1186/s13578-016-0109-3

**Published:** 2016-07-11

**Authors:** Yu Zhao, Hao Sun, Huating Wang

**Affiliations:** Li Ka Shing Institute of Health Sciences, The Chinese University of Hong Kong, Hong Kong, China; Department of Chemical Pathology, Prince of Wales Hospital, The Chinese University of Hong Kong, Hong Kong, China; Department of Orthopedics and Traumatology, Prince of Wales Hospital, The Chinese University of Hong Kong, Hong Kong, China

**Keywords:** lncRNAs, Dnmt, Methylation, Epigenetics

## Abstract

Long non-coding RNAs (lncRNAs) are being discovered as a novel family of regulators of gene expression at the epigenetic level. Emerging lines of evidence demonstrate that interplays between lncRNAs and DNA methylation machinery are an important layer of epigenetic regulation. Here in this mini-review we summarize the current findings in the field and focus particularly on the interactions mediated through direct physical association between lncRNAs and DNA methyltransferases (DNMTs).

## Background

DNA methylation is one of fundamental epigenetic mechanisms to regulate gene transcription, which involves the addition of methyl group to cytosines that are typically restricted to CpG dinucleotides [1]. The CpG-rich regions in genome, referred to as CpG islands, often occur at promoter and first-exon regions and are normally unmethylated; when they become methylated, the transcription of the cognate genes will be blocked [2, 3]. The establishment and maintenance of methylation patterns resulting in modulation of gene expression is one of the key steps in epigenetic regulation during normal development programs. Such process is precisely orchestrated by several DNA methyltransferases (DNMTs). In mammalian cells, DNMT3a and DNMT3b are known to de novo establish DNA methylation pattern whereas DNMT1 acts to maintain methylation status during DNA replication [4, 5]. It is now clear that dynamic changes in DNA methylation accompany development and other pathophysiological processes [6, 7]. For example, low methylation level was found at CpG-rich sequences at the pluripotency state of embryonic stem cells (ESCs); when differentiation processes ensue into the three germ layers, a global gain of DNA methylation occurs at the specific regions [7, 8]. In the absence of DNMTs, ESCs are competent for self-renewal but fail to initiate cellular differentiation [8–10]. Despite a wealth of knowledge on the dynamic changes of DNA methylation in diverse settings, there is still limited understanding of the mechanisms to determine the DNA regions targeted by DNMTs since they lack any sequence specificity other than CpG dinucleotides [4]. The interaction between histone modifiers and DNMTs provides one solution for the achieved sequence specificity. For example, EZH2 (a component of Polycomb Repressive Complex 2) was shown to interact with DNMTs and the presence of EZH2 is crucial for DNA methylation at EZH2-target promoters [11]. The H3K9 methyltransferase SETDB1 also directly associates with DNMT3a/b and their co-binding was observed at *RASSF1* promoter [12]. Recently, genome-wide DNA methylation profiles during cell differentiation have revealed the differentially methylated regions are enriched for transcription factor binding sites, implying potential roles of transcription factors in dictating the changes of DNA methylation landscape, which is supported by several pieces of experimental evidence [3, 13, 14]. For instance, E2F6 was found to recruit DNMT3B to a subset of germline gene promoters in somatic tissue, leading to the DNA methylation and the silenced state [15]. Notwithstanding these advances, key questions regarding how sequence-specific DNA methylation is orchestrated and whether additional players are involved remain largely unsolved. The novel family of regulators of gene expression, long non-coding RNAs (lncRNAs) seem to fill the gap.

**Fig. 1:**
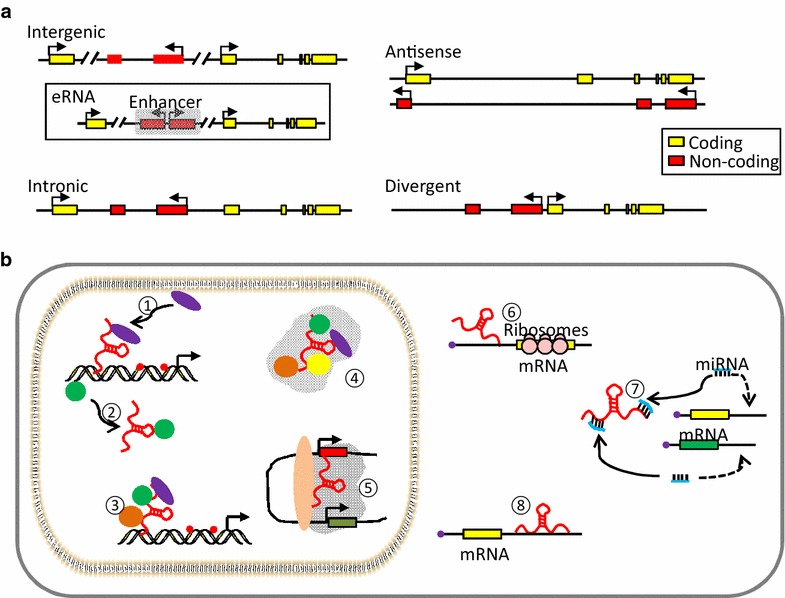
Genomic context and mechanisms of action of lncRNAs. **a** LncRNAs can be categorized according to their genomic loci relative to protein-coding genes. Intergenic lncRNAs (lincRNAs) are separate transcription units from protein-coding genes. Among them, a class may be transcribed from enhancers (eRNAs). Intronic lncRNAs are generated from the introns of protein-coding genes without overlapping with exons. Antisense lncRNAs are transcribed in opposite direction of protein-coding genes and overlap with the exons. Divergent lncRNAs are normally initiated from the promoter region of protein-coding genes. **b** LncRNAs exploit distinct mechanisms to elicit their regulatory roles in gene expression. ① lncRNAs can recruit proteins such as chromatin modifiers to target DNA; ② lncRNAs may act as decoy to titrate away DNA binding proteins like transcription factors; ③ lncRNAs can function as scaffold to bring multiple proteins into a complex; ④ to organize higher-order nuclear structure; ⑤ eRNAs can interact with Mediator and/or Cohesin complex to mediate and/or stabilize chromosomal looping between enhancers and cognate promoters. In cytoplasm, ⑥ lncRNAs can influence translation through intervening the loading of polysomes to mRNAs; ⑦ lncRNAs can serve as miRNA sponges to release their inhibitory roles on target mRNAs; ⑧ lncRNAs can regulate mRNA decay pathway, stabilizing or destabilizing mRNAs through RNA/RNA interaction with mRNA

A major advance in molecular biology over the past two decades has been the discovery and characterization of function for lncRNAs. The extraordinary maturation in sequencing technology, allowing the detection of low abundance transcripts in genome-wide transcriptomes analyses via massive parallel sequencing, has revealed significant levels of transcriptional activity within the unannotated and annotated regions of genome. Among many of the newly discovered non-coding transcripts, lncRNAs are the transcripts with a length over 200 nt, normally poorly conserved and do not serve as the templates for protein synthesis [16–19]. According to their genomic loci relative to protein-coding counterparts, lncRNAs can be further categorized as long intergenic ncRNAs (lincRNAs), antisense lncRNAs and intronic lncRNAs (Fig. 1) [16, 17]. Many of them are capped, spliced and polyadenylated similar to mRNAs. Recently, another class of lncRNAs, termed enhancer RNAs (eRNAs) generated from regulatory regions of genome, is emerging as important players in transcriptional activation of target mRNAs [20–22]. However, they are normally not spliced or polyadenylated. LncRNAs have been implicated in playing essential roles in every aspect of cellular processes and regulate gene expressions at different levels including chromatin organization, transcriptional control and post-transcriptional regulation (Fig. 1) [17]. The function of lncRNAs cannot currently be predicted from sequence information alone. An emerging theme, however, is the capacity of lncRNAs to modulate gene expression, with many of them participating in epigenetic control through interacting with various types of proteins involved in histone modification or chromatin remodeling. In particular, the Polycomb Repressive Complex 2 (PRC2), which is essential for embryonic development, binds numerous lncRNAs [23–25], fueling the idea that lncRNAs might be involved in targeting PRC2 to specific gene control elements. For instance, the lncRNA *Xist* (X-inactivation specific transcripts) binds to PRC2 to deposit repressive histone marks H3K27me3 along the X chromosome followed by inactivation of the marked copy, a mechanism required for dosage compensation in (mammalian) females [26, 27]. In addition to acting in epigenetic control through chromatin modification, emerging evidence has uncovered the underlying crosstalk between lncRNAs and DNA methylation. In this short review, we aim to summarize the relevant findings in this field emphasizing on those lncRNAs that have been identified to physically interact with DNMTs to regulate DNA methylation.

## ecCEBP

An outstanding example comes from a paper published in Nature [28], Di Ruscio and colleagues reported that a class of non-polyadenylated RNAs can regulate DNA methylation at a locus-specific pattern through interacting with DNMT1. In their findings, a nuclear-enriched lncRNA termed *ecCEBP* (extra-coding CEBPA) is expressed from *CEBPA* locus and shares a concordant expression pattern with *CEBPA* in human tissues. Silencing of this lncRNA diminishes the expression of *CEBPA*, suggesting its activating role. *CEBPA* is a known factor subject to the regulation of DNA methylation. Intriguingly, depletion of *ecCEBP* results in the increased methylation intensity at *CEBPA* promoter region. Moreover, overexpression of a region of *ecCEBP* is sufficient to alleviate the methylation level at the *CEBPA* locus and in turn increase the *CEBPA* expression. It is worthy to note that regulation of DNA methylation by *ecCEBP* is specific to the locus, since other genomic regions show little changes in DNA methylation pattern upon *ecCEBP* perturbation. Additionally, the authors demonstrated that *ecCEBPA* is physically associated with DNMT1; a subsequent detailed analysis revealed the DNMT1 with *ecCEBPA* RNA domains that assumed a stem-loop structure and mapped the RNA binding interface to a region encompassing the DNMT1 catalytic domain. Finally, the authors assessed the DNMT1-interacting RNAs at a genome-wide level by performing RIP followed by high throughput sequencing. Interestingly, they found an anti-correlation between DNMT1/RNA interaction and the methylation level at corresponding locus [28]. Taken together, this study has provided solid evidence for a model in which a class of non-polyadenylated lncRNAs regulate DNA methylation patterns through their association with DNMT1.

## Dali

Another example of DNMT1-interacting lncRNA comes from a recent publication from Chalei and colleagues [29]. In this study Chalei et al. revealed that a central nervous system-expressed lncRNA termed *Dali* is essential for neural differentiation and can regulate neural gene expression partially through interacting with DNMT1 to affect DNA methylation at distal target promoters. Notably, in this study the authors characterized *Dali*-associated DNA regions at a genome-wide level. They found *Dali* is associated with active chromatins revealed by the evidence that *Dali* bound regions are enriched for active histone marks like H3K4me3, H3K4me1 and H3K27ac. Additionally, the authors performed computational analysis to exclude the possibility that *Dali* directly interacts with DNA. On the contrary, they identified *Dali* interactions with DNMT1 to bring it to DNA regions; depletion of *Dali* increases DNA methylation at a subset of gene promoters, suggesting its inhibitory role in DNA methylation. Of note, the target promoters under *Dali* control are away from its site of transcription, indicating *Dali* modulates DNA methylation in trans. DNMT1 interacting transcription factors were thought to be required to target DNMT1 to specific loci. Interestingly, binding motifs of 9 such transcription factors are enriched in *Dali*-bound DNA regions, sparking a possibility that these factors determine the specificity of *Dali*-mediated DNA methylation change. These observations led to a model that lncRNA influences DNA methylation in trans at distal DNA regions through harnessing DNMT1 machinery [29].

## Dum

While the above studies were focused on DNMT1, the maintenance enzyme, other DNA methyltransferases enzymes may also associate with lncRNAs, which may modulate their enzymatic activity and the patterns of de novo DNA methylation. Indeed, a very recent report [30] from our group identified a lncRNA, *Dum* which coordinates the skeletal myoblast differentiation program through interacting with several DNMTs including DNMT1, DNMT3a and DNMT3b. In this work, we found *Dum* is induced by MyoD with an increased expression during differentiation process. Silencing of *Dum* impinges on myogenic differentiation in vitro and muscle regeneration in vivo, suggesting its pro-myogenic role. Additionally, *Dum* knockdown influenced the expression of several nearby genes, favoring a cis-action mode; and *Dppa2* was found to be a profound downstream target to recapitulate the roles of *Dum* in myogenesis. *Dppa2* was reported to be regulated by DNA methylation and our mechanistic studies revealed it is controlled by the interaction between *Dum* and the DNMTs. Depletion of *Dum* remarkably impaired the binding of DNMTs to the CpG regions of *Dppa2* promoter; while using chromatin isolation by RNA purification (ChIRP) assay, we retrieved *Dppa2* promoter regions, substantiating the indispensable role of *Dum* in DNMTs regulation on *Dppa2* promoter region. Of note, Dum/Dppa2 interaction requires the intra-chromosomal looping between these two loci. Cohesin components, NIPBL and RAD21, were shown to occupy both *Dppa2* and *Dum* regions and knockdown of the factors attenuated the repression of *Dppa2* expression, suggesting the long-range chromosomal interaction is required for the repressive role of *Dum* on *Dppa2* locus. Collectively, our study expands the concept of DNMT-interacting lncRNAs and suggests chromatin looping may be necessary to target the lncRNAs to particular loci [30].

## Dacor1

Besides these findings to dissect the connections between individual lncRNAs and DNMTs, it will be of great interest to identify the DNMT-interacting lncRNAs genome-widely. Indeed, the latest work done by Merry et al. [31] uncovered a total of 148 lncRNAs that are associated with DNMT1 in colon cancer cell through RNA immunoprecipitation sequencing (RIP-seq). Among them, the authors identified one lncRNA, referred to as *DACOR1* (DNMT1-associated Colon Cancer Repressed lncRNA 1), that is highly and specifically expressed in normal colon tissue while repressed in colon cancer cell lines. Intriguingly, overexpression of *DACOR1* in colon cancer cells resulted in a gain of DNA methylation at multiple loci without changing the DNMT1 expression level. Through ChIRP-seq (ChIRP sequencing) analysis, the authors demonstrated that *DACOR1* occupies a total of 338 genomic loci, of which 161 peaks are near 150 annotated genes. Notably, 31 of these sites overlapped with differentially methylated regions (DMRs) previously found in colon cancer samples versus matched normal tissues. These findings indicate that *DACOR1,* via its interaction with both chromatin and DNMT1, targets DNMT1 protein complex to the right genomic loci. Moreover, *DACOR1* was found to repress the expression of Cystathionine β-synthase (CBS) and in turn increase methionine, which is the substrate to produce S-adenosyl methionine (SAM). SAM is the key methyl donor for DNA methylation in mammalian cells. Thus, *DACOR1* may also impinge on DNA methylation through orchestrating cellular SAM levels [31].

## LincRNA-p21

In addition to the above examples of lncRNAs which regulate DNA methylation through physical association with DNMTs, many other lncRNAs may participate in modulating DNA methylation indirectly through interacting with other RNA binding proteins. A prominent example comes from a recent report from Bao and colleagues [32]. In their work, Bao et al. unraveled the mechanisms underlying *lincRNA*-*p21* functions in somatic cell reprogramming. They found *lincRNA*-*p21* is induced by p53 but not affect cell apoptosis or cell senescence. Instead, *lincRNA*-*p21* impedes cell reprogramming by maintaining the repressive histone mark H3K9me3 or DNA methylation at the promoters of different sets of pluripotent genes. Mechanistically, such actions are mediated via the interactions of *lincRNA*-*p21* with H3K9 methyltransferase SETDB1 and DNMT1 respectively, both of which are dependent on the RNA binding protein hnRNPk. Knockdown of hnRNPk attenuated the association between *lincRNA*-*p21* with SETDB1 or DNMT1 and in turn enhanced reprogramming efficiency. Hence, hnRNPk serves as a platform facilitating the connection between *lincRNA*-*p21* and DNMT1 to promote DNA methylation at specific loci [32].

## Other lncRNAs involved in DNA methylation

Another well-known lncRNA that affects DNA methylation is *Kcnq1ot1*, transcribed from the paternal allele of the imprinted *Kcnq1* locus. Besides the *Kcnq1* gene, this locus encodes eight other genes that are either ubiquitously or placental-specifically imprinted in a *Kcnq1ot1*-expression-dependent manner [33]. An 890 nt region close to the 5′ end of *Kcnq1ot1* is important for silencing and its genetic deletion in mice leads to abnormal silencing of ubiquitously imprinted genes, where a decrease in DNMT1 recruitment and subsequent reduction of CpG methylation occur [34]. However, a direct DNMT1–Kcnq1ot1 interaction has not been demonstrated. Additionally, a recently characterized lncRNA *PARTICLE* (promoter of *MAT2A*-antisense radiation-induced circulating lncRNA) [35] forms a DNA-lncRNA triplex upstream of a *MAT2A* promoter CpG island and represses *MAT2A* via methylation. Interestingly, *MAT2A* gene encodes the catalytic subunit of methionine adenosyltransferase which is an essential enzyme in methylation cycle. It means that lncRNA could interact with DNA methylation to regulate the expression of the methylation-modifying player, reflecting the close relationship between lncRNA and DNA methylation. Instead of showing the direct interaction between *PARTICLE* and DNMTs, the authors found that *PARTICLE* could interact with several chromatin suppressors such as G9a and SUZ12 (subunit of PRC2) thus serving as a recruiting platform for gene silencing complex. However, whether the methylation of *MAT2A* by *PARTICLE* was mediated by these chromatin suppressive proteins remains to be confirmed [35]. In addition to causing DNA methylation to silence gene expression, lncRNAs could also participate in demethylation leading to gene activation. For example, an antisense lncRNA, *TARID* (for *TCF21* antisense RNA inducing demethylation), could activates *TCF21* transcription by inducing demethylation of the *TCF21* promoter [36]. Mechanistically, it occurs through *TARID* binding to the *TCF21* promoter and recruiting GADD45A, which then recruits TDG together with TETs to direct base excision repair for demethylation.

## Conclusions

**Fig. 2:**
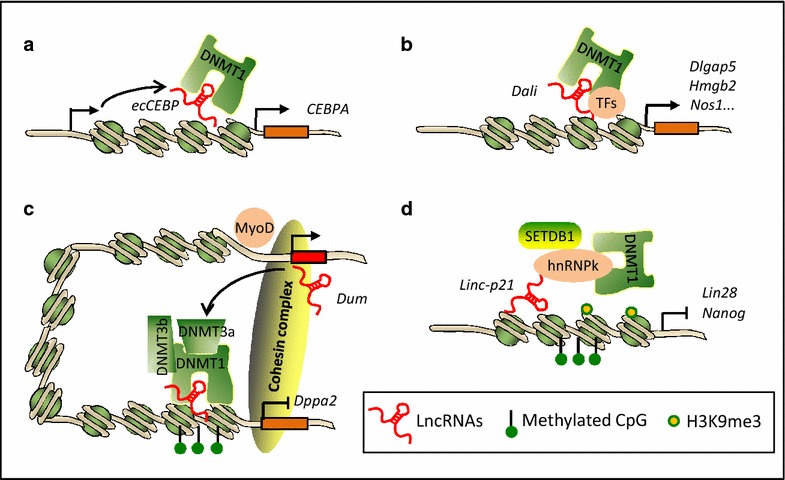
The regulatory mechanisms of DNA methylation by lncRNAs. **a** lncRNA/DNMT1 interaction prevents locus-specific DNA methylation locally *in cis* (for example, *ecCEBP*). **b** lncRNA/DNMT1 interaction modulates DNA-methylation at distant genomic loci in trans, which may also require DNMT1-associated transcription factors (TFs) (for example, *Dali*). **c** lncRNA interacts with all three DNMTs (DNMT1, DNMT3a and 3b) to mediate/maintain local DNA methylation at the target promoter (for example, *Dum*). **d** lncRNA interacts with DNMT1 indirectly through a protein intermediate (for example, *lincRNA*-*p21*)

In conclusion, as modeled in Fig. 2, lncRNAs can modulate the DNA methylation through interacting with diverse DNMT members directly or indirectly. Through the action of recruitment or eviction they may either promote or repress DNA methylation *in cis* or in trans. The dynamic nature of lncRNA repertoire as well as the RNA plasticity in interacting with diverse molecules such as DNA, RNA and protein renders lncRNA an ideal mediator to regulate local and sequence-specific DNA methylation or demethylation, hence resulting in global changes in DNA methylation profile, through which cells can respond to diverse stimuli. In the future we anticipate that many other lncRNAs will be discovered to be involved in DNA methylation control. The dysregulation of these lncRNAs may trigger epigenetic disorders in various human diseases. Therefore, the future efforts in studying the regulatory roles of lncRNAs in epigenetic mechanisms will open new avenues for therapeutic targets for DNA methylation processes.

## Abbreviations

CBS: cystathionine β-synthase
ChIRP: chromatin isolation by RNA purification
DACOR1: DNMT1-associated colon cancer repressed lncRNA 1
DNMT: DNA methyltransferase
ecCEBP: extra-coding CEBPA
eRNA: enhancer RNA
ESC: embryonic stem cell
lincRNA: long intergenic ncRNA
lncRNA: long noncoding RNA
PARTICLE: promoter of *MAT2A*-antisense radiation-induced circulating lncRNA
PRC2: polycomb repressive complex 2
RIP-seq: RNA immunoprecipitation sequencing
SAM: S-adenosyl methionine
TARID: TCF21 antisense RNA inducing demethylation
Xist: X-inactivation specific transcripts

## References

[1] Klose RJ, Bird AP (2006). Genomic DNA methylation: the mark and its mediators. Trends Biochem Sci.

[2] Lister R (2009). Human DNA methylomes at base resolution show widespread epigenomic differences. Nature.

[3] Ziller MJ (2013). Charting a dynamic DNA methylation landscape of the human genome. Nature.

[4] Shen H, Laird PW (2013). Interplay between the cancer genome and epigenome. Cell.

[5] Bedi U (2014). Epigenetic plasticity: a central regulator of epithelial-to-mesenchymal transition in cancer. Oncotarget.

[6] Xie W (2013). Epigenomic analysis of multilineage differentiation of human embryonic stem cells. Cell.

[7] Meissner A (2008). Genome-scale DNA methylation maps of pluripotent and differentiated cells. Nature.

[8] Fouse SD (2008). Promoter CpG methylation contributes to ES cell gene regulation in parallel with Oct4/Nanog, PcG complex and histone H3 K4/K27 trimethylation. Cell Stem Cell.

[9] Tsumura A (2006). Maintenance of self-renewal ability of mouse embryonic stem cells in the absence of DNA methyltransferases Dnmt1, Dnmt3a and Dnmt3b. Genes Cells.

[10] Jackson M (2004). Severe global DNA hypomethylation blocks differentiation and induces histone hyperacetylation in embryonic stem cells. Mol Cell Biol.

[11] Vire E (2006). The polycomb group protein EZH2 directly controls DNA methylation. Nature.

[12] Li H (2006). The histone methyltransferase SETDB1 and the DNA methyltransferase DNMT3A interact directly and localize to promoters silenced in cancer cells. J Biol Chem.

[13] Hogart A (2012). Genome-wide DNA methylation profiles in hematopoietic stem and progenitor cells reveal overrepresentation of ETS transcription factor binding sites. Genome Res.

[14] Feldmann A (2013). Transcription factor occupancy can mediate active turnover of DNA methylation at regulatory regions. PLoS Genet.

[15] Velasco G (2010). Dnmt3b recruitment through E2F6 transcriptional repressor mediates germ-line gene silencing in murine somatic tissues. Proc Natl Acad Sci USA.

[16] Rinn JL, Chang HY (2012). Genome regulation by long noncoding RNAs. Annu Rev Biochem.

[17] Kung JT, Colognori D, Lee JT (2013). Long noncoding RNAs: past, present and future. Genetics.

[18] Flynn RA, Chang HY (2014). Long noncoding RNAs in cell-fate programming and reprogramming. Cell Stem Cell.

[19] Fatica A, Bozzoni I (2014). Long non-coding RNAs: new players in cell differentiation and development. Nat Rev Genet.

[20] Lam MT (2014). Enhancer RNAs and regulated transcriptional programs. Trends Biochem Sci.

[21] Kim TK (2010). Widespread transcription at neuronal activity-regulated enhancers. Nature.

[22] Wang D (2011). Reprogramming transcription by distinct classes of enhancers functionally defined by eRNA. Nature.

[23] Zhao J (2010). Genome-wide identification of polycomb-associated RNAs by RIP-seq. Mol Cell.

[24] Khalil AM (2009). Many human large intergenic noncoding RNAs associate with chromatin-modifying complexes and affect gene expression. Proc Natl Acad Sci USA.

[25] Cifuentes-Rojas C (2014). Regulatory interactions between RNA and polycomb repressive complex 2. Mol Cell.

[26] Zhao J (2008). Polycomb proteins targeted by a short repeat RNA to the mouse X chromosome. Science.

[27] Jeon Y, Lee JT (2011). YY1 tethers Xist RNA to the inactive X nucleation center. Cell.

[28] Di Ruscio A (2013). DNMT1-interacting RNAs block gene-specific DNA methylation. Nature.

[29] Chalei V (2014). The long non-coding RNA Dali is an epigenetic regulator of neural differentiation. Elife.

[30] Wang LJ (2015). LncRNA Dum interacts with Dnmts to regulate Dppa2 expression during myogenic differentiation and muscle regeneration. Cell Res.

[31] Merry CR (2015). DNMT1-associated long non-coding RNAs regulate global gene expression and DNA methylation in colon cancer. Hum Mol Genet.

[32] Bao XC (2015). The p53-induced lincRNA-p21 derails somatic cell reprogramming by sustaining H3K9me3 and CpG methylation at pluripotency gene promoters. Cell Res.

[33] Mancini-Dinardo D (2006). Elongation of the Kcnq1ot1 transcript is required for genomic imprinting of neighboring genes. Genes Dev.

[34] Mohammad F (2010). Kcnq1ot1 noncoding RNA mediates transcriptional gene silencing by interacting with Dnmt1. Development.

[35] O’Leary VB (2015). PARTICLE, a triplex-forming long ncrna, regulates locus-specific methylation in response to low-dose irradiation. Cell Rep.

[36] Arab K (2014). Long noncoding RNA TARID directs demethylation and activation of the tumor suppressor TCF21 via GADD45A. Mol Cell.

